# Biofilm-Forming Antibiotic-Resistant Bacteria in Water From Distribution Systems: Occurrence and Public Health Implications

**DOI:** 10.1155/ijm/4147226

**Published:** 2024-11-27

**Authors:** Olorunjuwon O. Bello, Mathew O. Oni, Temitope K. Bello, Aderonke M. Ilemobayo, Adebanke M. Ajagunna, Adeleke Osho

**Affiliations:** ^1^Department of Microbiology, University of Medical Sciences, Ondo City, Nigeria; ^2^Department of Microbiology, Adeleke University, Ede, Osun, Nigeria; ^3^Department of Biological Sciences, Elizade University, Ilara-Mokin, Ondo State, Nigeria; ^4^Department of Microbiology, Redeemer's University, Ede, Osun State, Nigeria

**Keywords:** antibiotics, bacteria, biofilm, distribution system, public health, resistance, water

## Abstract

Biofilm is a structurally-connected microbial community, covered by a self-produced polymeric matrix and adhered to biotic or abiotic surfaces. This study aimed to evaluate the occurrence of biofilm-producing antibiotic-resistant bacteria in water from distribution systems. Water samples were taken from 32 tanks across Ondo City and Akure metropolis, Nigeria. Information regarding the sanitation status of the tanks was gathered by observation and oral interviews. The physicochemical properties were determined using standard methods. Using the pour plate technique. Agars included serially diluted water samples were inoculated onto plate count agar, mannitol salt agar, *Salmonella-Shigella* agar, MacConkey agar, and cetrimide nutrient agar to assess total viable bacteria, *Staphylococcus aureus*, *Salmonella* and *Shigella*, coliforms, and *Pseudomonas aeruginosa*, respectively. Eosin-methylene blue agar was used to cultivate *Escherichia coli* and *Enterobacter aerogenes.* Pure isolates were characterised using API kits and assessed for antibiotic resistance and biofilm production employing the Kirby-Bauer and tissue culture plate techniques, respectively. The ages of the water tanks ranged from 1 to 25 years old; all tanks had cover-lids; 13 (40.63%) had water guards while 12 (37.5%) underwent water treatment. The physicochemical properties chiefly fell within WHO standards for drinking water. One hundred and eighty-seven isolates were obtained. *S. aureus* (15.51%) had the highest frequency while *Salmonella enterica* (3.2%) had the lowest frequency. Thirty-six percent of the isolates were strong biofilm producers, while 20.67% Gram-negative and 18.69% Gram-positive bacterial isolates were antibiotic-resistant. This study revealed a high occurrence of biofilm-forming bacteria and prevalence of antibiotic-resistant bacteria in water distribution systems, emphasizing the urgency of improving water quality for public health protection.

## 1. Introduction

Many microbial species possess the ability to form biofilms and this process is often achieved by adhering to surfaces, excreting a slimy, and gummy substance to form a complex network on diverse surfaces. Bacteria, fungi, algae, protozoa and other microorganisms could converge to achieve the formation of biofilm. Biofilm formation begins with the attachment of microbial cells to biotic surface and followed with the aggregation of bacteria by cell-cell adhesion. This aggregation proceeds with the maturation of biofilm. Microorganisms in biofilms can include bacteria (including cocci, round, rod-shaped, filamentous and appendage bacteria). Biofilms on water pipes may be a complete film, or more commonly in water systems, small patches on pipe surfaces [1]. Biofilms in drinking water pipe networks can be responsible for a wide range of water quality and operational problems. Contamination of water within the distribution systems occurs as a result of the presence of biofilm-forming microbial species which attach and form on the walls of the water pipe and gets circulated within the water distribution systems. Numerous unwanted problems such as microbial contamination of the distributed water, biocorrosion, and lead-containing plumbing (in old networks) have been associated with the presence of biofilm [2].

Water is a limited natural resource and has remained an important substance to human health, food security, and the environment. Water and sanitation is the motive behind the sustainable development goal (SDG) 6. The goal clamours to make potable water available for all individuals irrespective of age, race, class or creed. Water is significant for overall development including health ecosystem, energy, human survival, socio-economic advancement and food production [3]. As the population of the world increases, there is a higher demand to balance the need for water resources in order that individuals have to access enough of the resources. The struggles for water are mostly triggered by the inability of households to meet water, agricultural, and industrial needs. Insufficient or lack of water disrupts the ecosystem and/or environmental services. The challenge of water supply and availability is diverse and pressing in many developing nations. This is a serious problem that necessitates the use of water storage systems in tanks to keep or preserve water for future use. This is a serious challenge in both rainy and dry seasons. The problem of water scarcity usually reaches its climax during the dry season while the rainy season normally avails few individuals the opportunity of insufficient water which is often unwholesome for consumption. This situation does not rule out the fact that some well-to-do individuals in many communities do have access to potable water but this would have cost them some luxuries to make such an amenity available at their doorsteps.

Water stored in tanks is often contaminated as a result of a lack of education or public enlightenment in relation to unhygienic domestic water handling practices, poor handling, and use of unclean containers, uncovered tanks, and natural contamination that occurred from the ambient domestic environment into the tanks. Any of these contamination processes could give room for interaction with the surface of the tanks and impair the distribution systems leading to biofilm formation. Biofilms could form on varieties of surfaces, living or nonliving, and the formation of bacterial biofilms is prevalent in hospital, industrial, and natural settings. The ability of microorganisms to form a connection of networks called biofilms, on diverse surfaces strengthens their growth and survival in environmental niches [4]. This strategy allows the microbial cells capable of forming this network to survive in harsh environmental conditions and also resist many antimicrobial agents. Every microbial biofilm community is very unique, even though some structural attributes can generally be considered universal. The aim of this study was to evaluate the public health potential risk associated with biofilm-producing antibiotic-resistant bacteria in stored water.

## 2. Materials and Methods

### 2.1. Description of Study Area

Ondo State is one of the states situated in southwest geo-political zone of Nigeria. Its population is presently estimated at 5,372,777. Ondo state covers 15,049 km^2^ with population density currently pegged at 410.3/km^3^ [5]. The University of Medical Sciences (UNIMED) is situated at Ondo city, one of the major cities of the state while the Federal University of Technology Akure (FUTA) is located in Akure, the capital of the state.

### 2.2. Assessment of the Sanitation Status of the Water Storage Tanks

The methods employed in gathering information regarding the sanitation status of the tanks typically involved a combination of observational assessments and oral interviews. Observation techniques included visually inspecting the physical condition of the tanks, assessing for any visible signs of biofilm formation, such as slimy layers or discoloration, and noting any potential sources of contamination in the surrounding environment. Oral interviews are conducted with individuals responsible for maintaining the water distribution systems and/or those with knowledge of the tank's sanitation practices. These interviews aim to gather qualitative data on the frequency and methods of tank cleaning, the use of disinfection agents, and any challenges or issues encountered in maintaining sanitation standards.

### 2.3. Collection of Water Samples

Water samples were collected from 32 storage tanks located at FUTA Health Centre (FH), Orita Obele (OO), Jibowu Hostel (JH), Saint Albert (SA), New-Town (NT), Kidney Care Centre (KC), UNIMED Odosida Campus (OC) and UNIMED Hostel (OH). All of these water storage tanks were placed on top of the scaffolding. The water samples were collected following standard protocols outlined by Prescott, Harley, and Klein [6]. Sampling was conducted from August 2022 to April 2023. To collect the samples, sterile containers were used to ensure the integrity of the samples and prevent contamination during collection. Samples were collected from various points within the water distribution system, including storage tanks, pipelines, and distribution points. After collection, the samples were immediately stored in a cool, dark environment to preserve their integrity before transportation to the laboratory for analysis. Proper chain of custody procedures was followed to maintain sample integrity and traceability throughout the process.

### 2.4. Physicochemical Analysis of Stored Water Samples Through Distribution Systems

Twelve essential parameters were considered for physicochemical water quality analysis and these were colour, temperature, turbidity, total dissolved solids (TDS), total suspended solids (TSS), pH, electrical conductivity (EC), nitrate ion (NO_3_^−^), chloride ion (Cl^−^) concentration, total alkalinity (TA), total hardness (TH) and dissolved oxygen (DO) concentration. The temperature, pH, and EC of the water samples were measured in situ with a mercury thermometer, glass electrode pH and EC metre, respectively. The TH of water was measured by titration with an EDTA and Eriochrome Black-T indicator [7]. The phenoldisulphonic acid colorimetric method was employed to determine the nitrate concentration; TA was assessed by CaCO_3_ through titration with a bromocresol green-methyl red indicator while the turbidity was evaluated using a turbidity metre. The Cl^−^ concentration was determined by the Mohr's method which entailed titrating the water sample with AgNO_3_ solution using potassium chromate as an indicator while the DO was assessed with a DO metre. The TSS was determined by filtration and subsequent oven-drying while the TDS concentration was determined with the TDS metre [7].

### 2.5. Bacteriological Examination of Stored Water Samples Through Distribution Systems

The isolation of possible microbial contaminants associated with the stored water was conducted within 8 days of storage. The information about the hygiene status was earlier provided by the personnel in charge of the storage tanks and distribution maintenance. For the examination of water samples from the intact storage tanks and distribution systems, a 200-mL portion of the stored water was collected in 300 mL sterile glass bottles and transported in an ice box to the laboratory. The microbiological analyses of water samples were carried out within 4 h of collection. A two-fold serial dilution was carried out on all water samples to reduce the bacterial concentration. Thereafter, using the pour plate technique, 0.5 mL of diluted water samples were inoculated on plate count agar, mannitol salt agar, *Salmonella-Shigella* agar, MacConkey agar, and cetrimide nutrient agar in duplicates, for the cultivation and enumeration of total viable bacteria, *Staphylococcus aureus*, *Salmonella* and *Shigella*, coliforms and *Pseudomonas aeruginosa*, respectively. *Escherichia coli* and *Enterobacter aerogenes* were cultivated on eosin-methylene blue agar. All plates were incubated at 37°C for 24 h. The discrete colonies were counted using a colony counter and counts expressed as colony-forming unit per mL (CFU/mL). Pure isolates were characterised based on cultural (colonial), microscopic and biochemical methods. The identities of the isolates were cross-matched with reference to standard manuals for the identification of bacteria. Several biochemical tests including catalase, coagulase, oxidase, hydrogen sulfide production, urease, indole, citrate utilization, methyl-red, Voges Proskauer, nitrate reduction, starch hydrolysis, gelatin hydrolysis and casein hydrolysis were carried out. The sugar fermentation tests carried out include glucose, sucrose, arabinose, maltose, xylose, galactose, sorbitol, inositol and raffinose tests. Further identification and confirmation of isolates were achieved by the API 20E and API 20NE were used for the confirmation of the family *Enterobacteriaceae* and non-*Enterobacteriaceae*, respectively [8].

### 2.6. Identification of Biofilm-Forming Bacterial Isolates

The tissue culture plate (gold-standard) method was employed for detection of biofilm-producing bacteria. Young actively-growing cultures were inoculated in 10 mL of trypticase soy broth containing 1% glucose and incubated at 37°C for 24 h. The cultures were then serially diluted to 10^−2^ with fresh medium. A 200-*μ*L quantity of the diluted cultures was introduced into each of the wells of sterile 96-well tissue culture plates and incubated at 37°C for 24 h. The wells with uninoculated sterile broth served as a negative control. After incubation, the contents of wells were discarded by subtle tapping, and wells were washed with 0.2 mL phosphate buffer saline with pH 7 to remove free-floating organisms. Two percent sodium acetate was applied to fix the biofilm formed by the adherence of bacteria to the wells and then stained with 0.1% crystal violet. Deionised water was used to eliminate excess stains before allowing the plates to dry up. The micro ELISA autoreader was used to evaluate the optical density (OD) of stained adherent biofilm at wavelength 570 nm. The experiment was repeated three times. The biofilm production was interpreted as stipulated by Stepanovic et al. [9] and Hassan et al. [10] (Table 1).

### 2.7. Determination of Resistance Patterns of the Bacterial Isolates to Antibiotics

The suspension of bacterial isolates (1.0 × 10^10^ CFU/mL^−1^), equivalent to 0.5 McFarland standard, was prepared. Briefly, 100 *μ*L of bacterial suspension was introduced into sterile normal saline to obtain the turbidity of 0.5 McFarland standard, which is a solution of barium sulphate prepared from 0.6 mL of 1% barium chloride added to 99.4 mL of sulphuric acid. The standard and the bacterial suspension were compared against a white background with contrasting black lines until the turbidity of the test organism matches with that of the standard. Each isolate was spread with a sterile swab stick on the entire surface of the agar plates. Then, a sterile forcep was used to place the antibiotics disc on the inoculated agar plates. The plates were incubated at 37°C for 48 h and antimicrobial activities were determined by measuring the zone of inhibition using a metre rule. The sensitivity patterns of the isolates were classified as susceptible (S), intermediately susceptible (I) and resistant (R), based on the measurements of the zones of inhibition (mm) in comparison to the guidelines of the Clinical Laboratory Standards Institutes [11]. However, only the percentage resistant profiles were reported. Gram-negative antibiotics discs (Oxoid) investigated were amikacin (30 *μ*g), amoxicillin (30 *μ*g), ampicillin (10 *μ*g), ceftazidime (30 *μ*g), cefazolin (30 *μ*g), ceftriaxone (30 *μ*g), chloramphenicol (30 *μ*g), clindamycin (2 *μ*g), gentamycin (10 *μ*g), imipenem (10 *μ*g), methicillin (5 *μ*g), netilmicin (30 *μ*g), ofloxacin (5 *μ*g) and piperacillin (100 *μ*g) while the Gram-positive antibiotic disks included amoxicillin (30 *μ*g), ampicillin (10 *μ*g), ceftazidime (30 *μ*g), ceftriaxone (30 *μ*g), ciprofloxacin (5 *μ*g), gentamycin (10 *μ*g), imipenem (10 *μ*g), linezolid (30 *μ*g), netilmicin (30 *μ*g), ofloxacin (5 *μ*g), penicillin (10 *μ*g), piperacillin (100 *μ*g), sulphamethazole (25 *μ*g), trimethoprim (5 *μ*g) and vancomycin (30 *μ*g).

### 2.8. Statistical Analysis

The data obtained from the physicochemical and bacteriological analyses of the water samples were compared using the one-sample *t*-test while one-way ANOVA was used to compare the data obtained from the different locations. The correlation analysis was run to determine the relationship between the presence of water coverlids, water guard, water treatments and microbial contaminations. The relationship between biofilm-production and antibiotics resistance of bacteria were also analysed. IBM SPSS Statistics Data Editor (Version 25) was used and the value of *α* was taken as 0.05.

## 3. Results and Discussion

Information regarding how long sampled tanks had been in use, when last the tanks were washed, use of cover lids and water treatments are shown in Table 2. Water samples were obtained from 32 different tanks whose ages ranged from 1 to 25 years. No reliable information was obtained to know when last twenty-four (75%) of the tanks were washed while it ranged from 8 to 12 months for the remaining tanks. All tanks from which water samples were taken had cover lids, 13 (40.63%) had water guards while 12 (37.5%) underwent water treatment.

The physicochemical properties and microbial loads of the investigated water are shown in Table 3. The pH of water ranged from 6.5 to 7.8 while temperature ranged from 27.1–29.8. The TDS, EC, colour, turbidity and NO_3_^−^ contents ranged from 204 to 565 mg/L, 103–427 µS/cm, 4–18 TCU, 2.8–6.2 NTU and 19.2–49.7 mg/L, respectively. The Cl^−^, TSS, TH, DO and TA ranged from 29.2 to 45.5 mg/L, 0.00–0.07 mg/L, 17.5–115.6 mg/L, 4.7–9.0 mg/L and 18.5–39.1 mg/L, respectively. The TVBC and TCC in water samples ranged from 2.2 × 10^2^–2.8 × 10^3^ CFU/mL and 0–1.8 × 10^2^ CFU/mL (Table 3).

**Figure 1:**
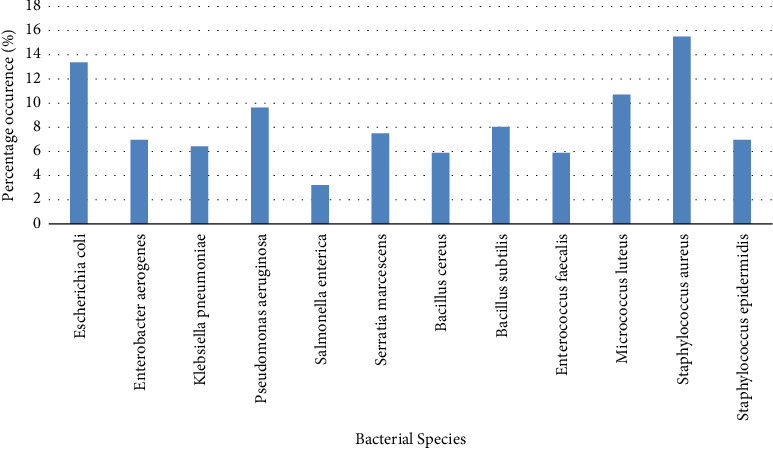
Percentage frequencies of bacteria in water samples obtained through the distribution systems from storage tanks.

One hundred and eighty-seven bacterial isolates were obtained from the water from the distribution systems and these were characterised as *Staphylococcus aureus* (15.51%) which had the highest percentage frequency, followed by *Escherichia coli* (13.38%), *Micrococcus luteus* (10.69%), *Pseudomonas aeruginosa* (9.63%), *Bacillus subtilis* (8.02%), *Serratia marcescens* (7.49%), *Enterobacter aerogenes* (6.95%), *Staphylococcus epidermidis* (6.95%), *Klebsiella pneumoniae* (6.42%), *Bacillus cereus* (5.88%) and *Enterococcus faecalis* (5.88%) while *Salmonella enterica* (3.2%) had the lowest frequency (Figure 1). More Gram-positive isolates (52.94%) were encountered as against Gram-negative isolates (47.06%). All *K. pneumoniae*, *P. aeruginosa* and *S. enterica* isolates were strong biofilm producers. Fourteen (56%) of the *E. coli* isolates, 12 (41.38%) of *S. aureus* and 5 (38.46%) of *S. epidermidis* exhibited strong biofilm-producing ability while other showed moderate strengths (Table 4).

**Figure 2:**
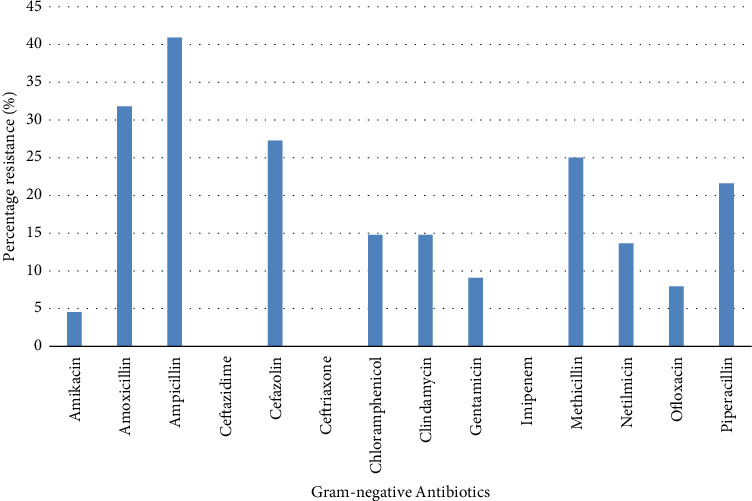
Percentage antibiotic resistance of Gram-negative bacterial isolates in water samples obtained through the distribution systems from storage tanks.

**Figure 3:**
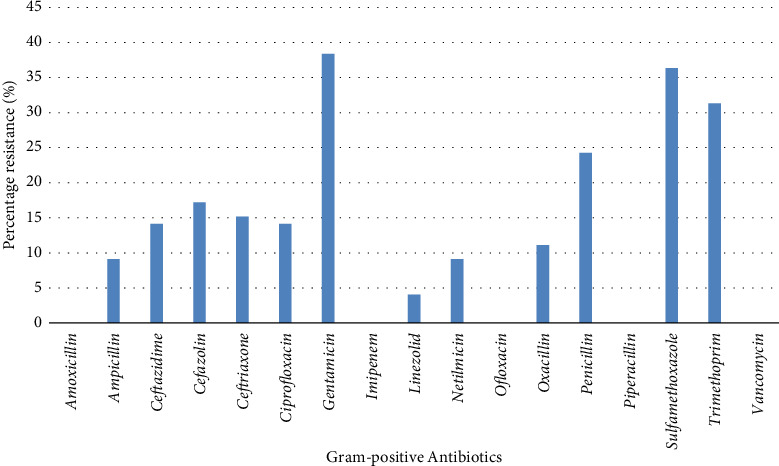
Percentage antibiotic resistance of Gram-positive bacterial isolates in water samples obtained through the distribution systems from storage tanks.

In all, 35.82% of bacterial isolates were strong biofilm producers, 39.57% showed moderate biofilm-producing strength while 24.60% were non/weak biofilm producers (Table 4). Amongst the 88 Gram-negative bacterial isolates, 36 (40.91%) exhibited resistance to ampicillin while all isolates showed susceptibility to ceftazidime, ceftriaxone and imipenem (Figure 2). Similarly, 38 (38.38%) of the 99 Gram-positive bacterial isolates elicited resistance to gentamicin while susceptibilities were shown to amoxicillin, imipenem, ofloxacin, piperacillin and vancomycin (Figure 3).

## 4. Discussion

The water in the water storage tanks, distributed though the water distribution systems, often get contaminated with microbial pathogens and this could be responsible for many cases of waterborne diseases; unfortunately, its incidence is usually underreported in this part of the world. Many households do not even take cognizance of the fact that there attitudes towards their storage tanks, distribution systems, hygiene practices and water purification processes could influence the incidence of waterborne diseases. Unfortunately, many waterborne diseases are not usually traced to their aetiology and this could not be dissociated from the high level of ignorance and unawareness of the role the water storage tanks and the distribution systems play in the dissemination of antibiotic-resistant pathogens that are even capable of producing biofilms. This is, indeed, a neglected public health threat which requires multidimensional approaches. There was positive correlation between presence of cover lids, use of water guards, water treatments and the microbial loads in the water samples (*r* = 0.97). However, there was no statistical relationship between the ages of the storage tanks and microbial contamination (*r* = 0.31). The information in relation to these determinants is shown in Table 2. This position was supported by the findings of Chalchisa, Megersa and Beyene [12] who reported that water storage tanks was one of the major causes of waterborne diseases.

Determining the physicochemical constituents of water is crucial water quality assessment. A safe and wholesome water is clear, colourless, odourless and tasteless. No water is considered safe for drinking or cooking if it appears turbid and a turbid water will produce odour and taste which could be simply attributed to the activities of microorganisms and presence of mineral matter and chemical substances. In water from distribution systems, this state could be connected to corrosion of water pipes or as a result of water treatment [13]. All water samples investigated in this study had no objectionable colour, odour or taste.

Cl^−^ occurs in most natural water but its concentration in water considered for drinking should not exceed 250 mg/L. The Cl^−^ concentration in the studied water ranged from 29.2 to 45.5 mg/L and fell within the recommended value. The EC predicts the ion concentrations or total dissolved salts in water. The movement of ions in solution form aids the conduction of electric current [14]. The permissible limit for EC is 750 *μ*S/cm [13] and it is relevant in the assessment of the purity of water. The EC of the collected samples ranged from 103–427 µS/cm. The TDS are usually contaminants in water and its range in this study was from 204 to 565 mg/L. These parameters were within the WHO recommended standards. Dissimilar ranges were reported by Chalchisa et al. [12] and Mohamed et al. [15] as a few of their values deviated from the permissible limits. Nitrate measures the oxidised and stable form of nitrogen in a water body. A high concentration of nitrates reflects the status of micronutrients in the water and this could support microbial and plant growths in the distribution system. The NO_3_^−^ contents ranged from 19.2 to 49.7 mg/L which was within the WHO recommended standards of < 50. Although, Chigor et al. [16] reported that nitrate concentration exceeding 10 mg/L in drinking water could cause high mortality in infants. This assertion has not been confirmed elsewhere to the best of our knowledge. However, the results of this study agree with the reports of Duressa, Assefa, Jida [17] and Adesakin et al. [18].

The pH reflects the relative acidity or alkalinity and measures the active hydrogen ion (H^+^) concentration in the water. Water that ranged from 6.5 to 8.5 are good for drinking recommended and standards by World Health Organization (WHO) and Standard Organization of Nigeria (SON). The pH of water investigated in this study ranged from 6.5 to 7.0. These values generally fell within the WHO standard for drinking. Temperature values recorded in the water samples ranged from 26.9°C to 29.8°C. These temperatures could have encouraged the proliferation of biofilm-producing bacteria in the water distribution systems. Total alkalinity evaluates the buffering capacities of water. The alkalinity of water samples ranged from 18.1 to 39.5 mg/L and these were generally within the limits of WHO [13]. Water with high alkalinity is not wholesome and this could be associated with the state of the distribution pipes, and biofilm production by certain bacteria.

The notion of coliforms as bacterial indicators of the microbial quality of water is primarily based on the fact that coliforms are found in excessive numbers in the faeces of humans and other warm-blooded animals. Thus, their presence suggests contamination with the matter of faecal origin [19]. The bacteria isolated were *S. aureus*, *E. coli*, *M. luteus*, *P. aeruginosa*, *B. subtilis*, *S. marcescens*, *E. aerogenes*, *S. epidermidis*, *K. pneumoniae*, *B. cereus*, *E. faecalis* and *S. enterica*. All water samples investigated had the presence of faecal pollution. And which indicated that indicated that the waters were unwholesome for human consumption and should undergo thorough treatment before drinking. The occurrence of these bacteria could be linked to improper handling, dirty storage tanks, corroded pipes, poor purification procedures and unhygienic practices. This is consistent with the reports of Agwu and Avoaja [20]; Bello et al. [21]; Adesakin et al. [18]; Ondieki et al. [22] and Mohamed et al. [15].

*E. coli* is conventionally inhabitant the intestines of most man and animals. It causes bacterial intestinal and extra-intestinal infections including septicaemia, diarrhoea, urinary tract infections (UTIs) and neonatal meningitis in man and livestock [23]. *P. aeruginosa* causes infections in immune-compromised but hardly establish infections in healthy individuals [24]. It has been labelled amongst the top-10 hospital “superbugs” as a result of its widespread antimicrobial-resistant strains leading to life-threatening health conditions [25]. It is a potent biofilm-producer which acts as a barrier in wound healing process, and it displays high resistance to antimicrobials [26, 27]. *C. freundii* is commonly found in soil, and water. The organism is also associated with nonpotable water that normally undergo poor chlorination process and its mode of transmission could be either by direct contact or droplets or through faecal-oral route, but person to person transmission is more prevalent [28]. *E. aerogenes* can, by nature, be aerobic or anaerobic. It causes infections such as bacteremia, endocarditis, septic arthritis, skin and soft-tissue infections, lower respiratory tract infections, UTIs, osteomyelitis, CNS infections, intra-abdominal infections and ophthalmic infections [29].

*S. aureus* is an opportunistic pathogens and have been linked with high mortality, especially if not effectively treated [30]. The bacterium is resistant to many conventional antibiotics and has recently developed resistance to daptomycin and linezolid which are the last line drugs [8]. *K. pneumoniae* could be found in surface waters, soil, plants and intestines of man and animals. It causes infections such as pneumonia, UTIs and bloodstream infections, in immunocompromised individuals. *K. pneumoniae* infections are threatening to public health because of its acquisition of multidrug-resistant (MDR) genes [31]. The presence of *S. enterica* in water is indicative of pollution with matter of faecal origin. Salmonellae resides the gastrointestinal tracts of man and animals. Salmonellosis is regarded as a water- and food-borne infection and has to have high incidence rates. The organism is transmitted through faecal-oral route [32]. *Bacilli* are often detected in drinking water supplies, and this could be largely connected to the resistance of spores to disinfection processes.

It was already noted that 36% of the bacterial isolates were strong-biofilm producers while 39% were moderate biofilm-producers (Figure 2). Relatively high percentages of the Gram-negative bacterial isolates exhibited resistance to ampicillin, amoxicillin, cefazolin, methicillin and piperacillin (Figure 2). Similarly, the Gram-positive isolates elicited high resistance to gentamicin, sulphamethoxazole, trimethoprim and penicillin (Figure 3). Despite the fact that more Gram-positive isolates were encountered in the water samples, a higher percentage of Gram-negative bacteria elicited resistance to the investigated antibiotics than the Gram-positive bacteria. It was noted that all strong biofilm-producers and a high number of moderate biofilm-producers were resistant to two or more classes of the antibiotics, and this indicted them as MDR bacteria. The relationship between biofilm production and bacterial resistance was significant (*p*=0.04). This finding was buttressed by Qi et al. [33] who reported the relationship between antibiotic resistance, biofilm formation and biofilm-specific resistance in a bacterium. Wang et al. [34] observed that the occurrence and fate of antibiotic resistant genes and antibiotic resistant bacteria in municipal wastewater treatment plant is associated with hygiene and sanitation. These findings were further buttressed by Gebreyohannes, Nyerere, Bii [35] and Brown et al. [36]. A high percentage of death in developing countries is associated with unhygienic water and sanitation, and untreated distribution systems could be a veritable reservoir of several other opportunistic pathogens, and chemical poisoning [37, 38].

## 5. Conclusion

This study revealed a high occurrence of biofilm-forming bacteria and prevalence of antibiotic-resistant bacteria in water distribution systems, emphasizing the urgency of improving water quality for public health protection. It showed that some unhygienic practices, such as the absence of coverlids, nonusage of water guards and lack of water treatments, could lead to high microbial contamination of the water obtained from the storage tanks through the distribution systems. Most water samples contained a diverse group of bacteria that exhibited resistance to two or more classes of antibiotics, including several viable potential pathogenic species which are of public health concerns. It is recommended that environmental agencies and water management boards carry out a periodical enlightenment for the people to inform and educate them on how to maintain a clean water storage system and on how they can reduce the risk of biofilms and MDR microorganisms in their drinking water distribution system. Also, chlorine and other water protection kits should be made readily available and circulated at very pocket friendly prices.

## Figures and Tables

**Table 1:** Interpretation of biofilm production in isolates from water storage tanks.

Biofilm production	Average OD value
Non/weak	≤ ODc/ODc < ≤ 2*x* ODc
Moderate	2*x* ODc <–≤ 4*x* ODc
Strong	> 4*x* ODc

*Note:* ODc = optical density of stained adherent biofilm.

**Table 2:** Information on the storage tanks from which water samples were collected.

Sample codes	Age of tanks (Year)	Last washed	Presence of cover lids	Presence of water guards	Water treatment
FH1	25	Unknown	Yes	No	No
FH2	15	Unknown	Yes	No	No
FH3	10	Unknown	Yes	No	No
FH4	25	Unknown	Yes	No	No
OO1	10	8 months	Yes	Yes	Yes
OO2	18	10 months	Yes	Yes	Yes
OO3	12	8 months	Yes	Yes	Yes
OO4	12	12 months	Yes	Yes	Yes
JH1	1	Unknown	Yes	Yes	Yes
JH2	2	Unknown	Yes	No	Yes
JH3	2	Unknown	Yes	No	Yes
JH4	1	Unknown	Yes	Yes	Yes
SA1	2	10 months	Yes	Yes	Yes
SA2	4	7 months	Yes	Yes	Yes
SA3	2	7 months	Yes	Yes	Yes
SA4	10	10 months	Yes	Yes	Yes
NT1	8	Unknown	Yes	Yes	No
NT2	15	Unknown	Yes	No	No
NT3	6	Unknown	Yes	Yes	No
NT4	3	Unknown	Yes	Yes	No
KC1	9	Unknown	Yes	No	No
KC2	6	Unknown	Yes	No	No
KC3	9	Unknown	Yes	No	No
KC4	6	Unknown	Yes	No	No
OC1	7	Unknown	Yes	No	No
OC2	7	Unknown	Yes	No	No
OC3	6	Unknown	Yes	No	No
OC4	6	Unknown	Yes	No	No
OH1	5	Unknown	Yes	No	No
OH2	5	Unknown	Yes	No	No
OH3	5	Unknown	Yes	No	No
OH4	5	Unknown	Yes	No	No

**Table 3:** The physicochemical properties and microbial loads in water samples obtained through the distribution systems from storage tanks.

Code	pH	Temp	TDS (mg/L)	EC (*μ*S/cm)	Colour TCU	Turbidity	NO_3_^−^	Cl^−^	TSS	TH	DO	TA	TVBC × CFU/mL	TCC × 10^2^ CFU/mL
		°C				NTU	mg/L	mg/L	mg/L	mg/L	mg/L	mg/L		
WHO (1996)	6.5–8.5	Ambient	< 500	< 400	< 15	< 5	< 50	< 250	None	See footnote	None	< 75	0 CFU/mL	0 CFU/100 mL
FH1	6.7	29.6	454^c^	182^a^	15^b^	4.2	45.3^d^	36.8^a^	0.01	20.8^a^	5.3	21.7^a^	1.1 × 10^3d^	1.1
FH2	7	29.3	365^b^	211^b^	8^a^	3.9	37.5^c^	35.2^a^	0	31.2^b^	5.1	35.4^c^	1.8 × 10^2a^	0
FH3	6.8	29.6	318^b^	197^b^	11^b^	4.5	45.2^d^	34.3^a^	0	28.9^a^	5.3	28.1^b^	9.0 × 10^2c^	1.1
FH4	7.4	29.6	478^c^	176^a^	7^a^	4.9	33.5^c^	41.1^b^	0.01	39.1^b^	5.9	19.8^a^	8.8 × 10^2c^	1.2
OO1	7.7	29.8	394^a^	103^a^	9^a^	3.5	38.2^c^	40.4^b^	0.03	68.4^e^	9	18.5^a^	9.0 × 10^2c^	1
OO2	6.9	28.4	509^d^	218^b^	5^a^	4	40.5^d^	32.2^a^	0.01	71.6^e^	5.9	20.6^a^	9.5 × 10^2c^	1.2
OO3	7.8	28.3	204^a^	201^a^	13	3.9	43.5^d^	41.1^b^	0.01	43.9^c^	7.4	25.5^b^	1.3 × 10^3d^	1.8
OO4	7.5	28.5	417^c^	171^a^	12^b^	5.3	45.3^d^	30.2^a^	0.02	63.5^e^	5.5	19.2^a^	9.8 × 10^2c^	1.2
JH1	7.5	28.4	413^c^	222^b^	4^a^	4.5	43.5^d^	48.2^b^	0.04	87.1^f^	5.7	30.4^c^	8.9 × 10^2c^	1.1
JH2	7.3	28.3	417^c^	315^c^	12^b^	5.3	49.7^d^	32.3^a^	0.02	64.8^e^	7.9	37.8^c^	1.2 × 10^3d^	1.2
JH3	7.4	29.3	586^d^	283^b^	9^a^	4.7	40.5^d^	31.4^a^	0.01	85.3^f^	7.5	23.8^b^	1.1 × 10^3d^	1.5
JH4	7	28.2	485^c^	247^b^	5^a^	3.1	36.6^c^	41.1^b^	0.02	69.9^e^	6.9	39.5^c^	9.5 × 10^2c^	0
SA1	6.5	28.3	418^c^	351^c^	18^b^	6.2	38.9^c^	40.0^b^	0.03	61.4^e^	7.5	22.4^b^	7.0 × 10^2c^	0
SA2	7	29.2	385^b^	298^c^	13^b^	4.9	35.5^c^	38.2^a^	0.02	99.6^g^	6	38.5^c^	2.8 × 10^3d^	1.5
SA3	7.2	29.4	365^b^	323^c^	6^a^	3	34.4^c^	38.3^a^	0.01	78.7^f^	5.9	27.7^b^	2.6 × 10^3d^	1.2
SA4	7.1	29.7	413^c^	306^c^	10^b^	3.8	35.9^c^	44.5^b^	0.03	101.6^g^	5.3	20.3^b^	1.1 × 10^3d^	1.5
NT1	6.7	28.5	565^d^	427^d^	15^b^	5	47.2	46.8^b^	0.03	96.4^g^	8.1	19.6^a^	1.0 × 10^3d^	1
NT2	7	27.8	462^c^	396^d^	12^b^	4.1	43.2	45.2^b^	0.07	115.6^h^	6.6	28.5^b^	1.1 × 10^3d^	1.5
NT3	6.8	28.6	437^c^	357^d^	17^b^	5.5	45.1^d^	44.3^b^	0.04	99.1^g^	4.7	18.1^a^	1.0 × 10^3d^	1
NT4	7.4	27.2	529^d^	258^b^	12^b^	3.9	43.5^d^	41.2^b^	0.03	108.2^h^	6.3	36.2^c^	5.6 × 10^2b^	0
KC1	6.7	26.9	421^c^	257^b^	13^b^	4.7	45.8^d^	40.6^b^	0	20.9^a^	5.5	39.1^c^	8.8 × 10^2c^	1.2
KC2	6.9	28.4	385^b^	278^b^	12^b^	4.5	48.5^d^	45.5^b^	0	17.5^a^	5.9	35.8^c^	9.0 × 10^2c^	1.1
KC3	7.8	28.6	369^b^	301^c^	16^b^	4.9	15.3^a^	31.1^a^	0	22.2^a^	6	37.9^c^	9.2 × 10^2c^	0
KC4	6.5	29.2	401^c^	290^c^	10^b^	3.8	40.5^d^	35.2^a^	0	35.1^b^	5.8	31.9^c^	9.2 × 10^2c^	0.5
OC1	6.5	27.7	354^b^	335^c^	7^a^	3.2	23.5^b^	38.2^a^	0.03	42.6^c^	7.5	37.7^c^	7.8 × 10^2b^	1.2
OC2	7.3	27.3	321^b^	396	11^b^	4	27.7^b^	32.3^a^	0.01	36.9^b^	6.1	35.9^c^	2.2 × 10^2a^	1.2
OC3	7.4	29.1	289^a^	257^b^	9^a^	3.7	19.2^a^	31.5^a^	0.05	57.1^d^	7.1	37.4^c^	2.5 × 10^2a^	1
OC4	7	29.5	303^b^	365^c^	7^a^	3.5	31.5^c^	41.1^b^	0.03	48.3^c^	6.9	33.5^c^	7.7 × 10^2b^	1.3
OH1	6.5	29.2	432^c^	297^c^	14^b^	4.6	27.8^b^	34.0^a^	0.01	57.0^d^	5.9	25.7^b^	1.1 × 10^3d^	1
OH2	7	29.5	377^b^	372^c^	6^a^	2.8	23.7^b^	38.2^a^	0.01	49.8^c^	4.5	29.2^b^	3.9 × 10^2a^	1.5
OH3	7.2	27.1	338^a^	251^b^	9^a^	3.2	30.7^c^	33.7^a^	0.01	51.7^d^	5.3	34.9^c^	7.7 × 10^2b^	1.1
OH4	7.1	29.3	366^a^	281^b^	13^b^	4.1	38.5^c^	29.2^a^	0.01	45.5^c^	6.1	28.6^b^	2.2 × 10^2a^	0

*Note:* Values with same superscript showed no significant difference taking *α* to be equal to 0.05. Each value represents the mean of triplicate samples. (The WHO grades drinking water as soft if values are below 17.1 mg/L), slightly hard (with values between 17.1 and 60 mg/L), moderately hard (with values between 60 and 120 mg/L), hard (with values between 120 and 180 mg/L) and very hard (values greater than 180 mg/L).

Abbreviations: Cl^−^ = chloride ion concentration, DO = dissolved oxygen, EC = electrical conductivity, TA = total alkalinity, TCC = total coliform count, TH = total hardness, TSS = total suspended solids, TVBC = total viable bacterial count.

**Table 4:** Percentage occurrence and biofilm strength of biofilm-producing bacterial species in water samples obtained through the distribution systems from storage tanks.

Bacteria		*N*	Number of isolates with biofilm-producing potentials (*n* %)		
			Strong (%)	Moderate (%)	Non/weak (%)
Gram negative bacteria	*Escherichia coli*	25	14 (56)	11 (44)	0 (0)
	*Enterobacter aerogenes*	13	0 (0)	8 (61.54)	5 (38.46)
	*Klebsiella pneumoniae*	12	12 (100)	0 (0)	0 (0)
	*Pseudomonas aeruginosa*	18	18 (100)	0 (0)	0 (0)
	*Salmonella enterica*	6	6 (100)	0 (0)	0 (0)
	*Serratia marcescens*	14	0 (0)	5 (35.71)	9 (64.29)

	Total Gram-negative bacteria (%)	88	50 (56.82)	24 (27.27)	14 (15.91)

Gram-positive bacteria	*Bacillus cereus*	11	0 (0)	5 (45.45)	6 (54.55)
	*Bacillus subtilis*	15	0 (0)	9 (60)	6 (40)
	*Enterococcus faecalis*	11	0 (0)	4 (36.36)	7 (63.64)
	*Micrococcus luteus*	20	0 (0)	7 (35)	13 (65)
	*Staphylococcus aureus*	29	12 (41.38)	17 (58.62)	0 (0)
	*Staphylococcus epidermidis*	13	5 (38.46)	8 (61.54)	0 (0)

	Total Gram-positive bacteria (%)	99	17 (17.17)	50 (50.50)	32 (32.32)

	*Total bacteria (%)*	187	67 (35.82)	74 (39.57)	46 (24.60)

## Data Availability

All data generated and analysed during this study are included in this article.
